# Periodontal Disease-Induced Atherosclerosis and Oxidative Stress

**DOI:** 10.3390/antiox4030577

**Published:** 2015-09-02

**Authors:** Tomoko Kurita-Ochiai, Ru Jia, Yu Cai, Yohei Yamaguchi, Masafumi Yamamoto

**Affiliations:** 1 Department of Microbiology and Immunology, Nihon University School of Dentistry at Matsudo, Chiba 271-8587, Japan; E-Mail: yamamoto.rika@nihon-u.ac.jp; 2 Stomatology Hospital, Tongji University, Shanghai 200072, China; E-Mail: ru_jia@hotmail.com; 3 Department of Periodontology, Peking University School and Hospital of Stomatology, 22 Zhongguancun Avenue South, Haidian District, Beijing 100081, China; E-Mail: jessonjesson@hotmail.com; 4 Department of Oral and Maxillofacial Surgery, School of Medicine, Tokyo Women’s Medical University, Tokyo 162-8666, Japan; E-Mail: yamayo030783@yahoo.co.jp

**Keywords:** inflammation, oxidative stress, systemic chronic disease, atherosclerosis, periodontal disease

## Abstract

Periodontal disease is a highly prevalent disorder affecting up to 80% of the global population. Recent epidemiological studies have shown an association between periodontal disease and cardiovascular disease, as oxidative stress plays an important role in chronic inflammatory diseases such as periodontal disease and cardiovascular disease. In this review, we focus on the mechanisms by which periodontopathic bacteria cause chronic inflammation through the enhancement of oxidative stress and accelerate cardiovascular disease. Furthermore, we comment on the antioxidative activity of catechin in atherosclerosis accelerated by periodontitis.

## 1. Introduction

Periodontal disease, a chronic, destructive, and inflammatory condition affecting a large portion of the adult population, is a major cause of tooth loss and is characterized by a chronic infection associated with gram-negative anaerobic bacteria in the dental biofilm. It leads to the irreversible destruction of tissues supporting the teeth and is clinically detectable as periodontal pockets and alveolar bone loss [1,2]. Because periodontitis often causes bacteremia, a relationship between periodontitis and systemic diseases via periodontal pathogens has been explored [3]. Two or more periodontopathic bacteria have been detected in the cardiac valve [4,5] and in aortic aneurysm cases [6]; therefore, periodontal infection may also affect the progression of cardiovascular disease (CVD). However, many questions regarding this causal relationship and its pathological mechanism remain unanswered. This review discusses the involvement of oxidative stress caused by periodontopathic bacteria in the development of atherosclerosis. Furthermore, we mention the possibility of preventing atherosclerosis by the intake of catechin, which has both antioxidative and anti-inflammatory effects.

## 2. Increase in Oxidative Stress and Lipid Oxidation Caused by Infection

Periodontitis is a multifactorial disease, and the genetic background of patients as well as the presence of pathogenic bacteria and the immune mechanisms are thus important elements. The occurrence and progress of inflammatory responses in the periodontium are a result of the interaction between the bacteria that exists in the subgingival space and the immune cells in periodontal tissue that respond to them.

It is said that periodontopathogens injure tissue directly through harmful toxic products that induce cell death and tissue necrosis and that they do so indirectly through the activation of inflammatory cells that produce inflammatory cytokines [7]. However, many microbial products have little or no direct toxic effect on the host. Instead, they possess the potential to activate inflammatory reactions that cause tissue damage. It is now well accepted that it is the host’s response to the plaque bacteria, rather than microbial virulence *per se*, that directly causes the tissue damage. During the course of inflammation in response to dental plaque bacterial species, the gingival fibroblasts produce interleukin-1 (IL-1), IL-6, IL-8, tumor necrosis factor-a (TNF-α), and transforming growth factor beta (TGF-β) [8,9,10]. These cytokines and chemokines are an important mediator of the inflammatory responses, and some of them recruit polymorphonuclear leukocytes to the site of infection [11], which then produce reactive oxygen species (ROS) through NADPH oxidase catalyzation [12]. Oxidative DNA damage in fibroblasts and increased ROS production in polymorphonuclear leukocytes have been reported in experimental periodontitis in animal studies [13,14]. Patients with periodontitis also have increased levels of lipid peroxidation in plasma, saliva, and gingival crevicular fluid [15], and these levels have been correlated with the severity of periodontal disease [15,16]. Furthermore, the inflammatory response stimulates increased osteoclastic bone resorption, which degrades the alveolar bone supporting the teeth in the infected area. Overwhelming evidence indicates that oxidative stress plays a central role in the periodontal tissue and alveolar bone destruction [17,18]. Indeed, a recent clinical study indicated that an increase in plasma reactive oxygen metabolites (ROM) levels was associated with that in the clinical attachment level in periodontal patients whose plasma ROM level was low [19].

Mitochondria are the major producer of ROS in cells, and disruption of the structure and function of mitochondria are now thought to trigger numerous diseases such as atherosclerotic cardiovascular disease, obesity and type2 diabetes [20]. Chronic over-production of mitochondrial reactive oxygen species leads to increased oxidation of low-density lipoprotein (LDL) in atherosclerosis [21]. Therefore, lipid peroxidation also plays an important role in cardiovascular diseases [22]. In particular, LDL oxidation may be a key step in the development of atherosclerosis [23]. Oxidation of LDL is essential for its accumulation within macrophages and the formation of foam cells, which can upregulate proatherogenic chemokines and adhesion molecules [24] and induce IL-6, TNF-α, and C-reactive protein secretion [25]. *Aggregatibacter actinomycetemcomitans* infection of apolipoprotein E-deficient spontaneously hyperlipidemic (ApoE^shl^) mice promoted LDL oxidation, as indicated by marked upregulation of 4-hydroxynoneal, oxidated LDL (ox-LDL), and phospholipase A2 in the aorta; ox-LDL, 8-oxo-2′-deoxyguanosine, and myeloperoxidase serum levels; and aortic expression of nicotinamide adenine dinucleotide phosphate oxidase, caveolin-1, and the receptor for advanced glycation endproducts (RAGE) [26]. *Porphyromonas gingivalis* also increased oxidative modification of LDL [27,28] and rupture of atherosclerotic plaque through induction of matrix metalloproteinase (MMP) [29]. Co-incubation of a murine macrophage cell line with *P. gingivalis* in the presence of LDL resulted in the formation of foam cells in a dose-dependent manner [30].

These results suggest that periodontopathic bacteria contribute to lipid oxidation and further inflammatory induction in remote structures as well as the oral cavity.

## 3. Activation of the Inflammasome in Periodontal Disease and Atherosclerosis

Although the secretion of cytokines at inflammatory sites is initially protective in the elimination of infectious bacteria, excessive or sustained production of proinflammatory cytokines is related to periodontal destruction, with periodontal attachment loss and alveolar bone resorption. The cytokine concentration in gingival crevicular fluid (GCF) and saliva is higher in patients with aggressive periodontitis than in healthy subjects and decreases after periodontal treatment. [31,32]. Macrophages represent an important source of pro-inflammatory cytokines, including IL-1β and TNF-α; under dysregulation, these contribute to the destruction of host tissue. Thus, understanding the mechanisms of periodontal pathogen-induced immune cell signaling could provide useful information for the prevention and treatment of periodontitis.

*P. gingivalis* is a gram-negative asaccharolytic bacterium that has long been implicated in human periodontitis [33]. Recent evidence suggests that this bacterium contributes to periodontitis by functioning as a keystone pathogen [34,35]. Virulence factors of *P. gingivalis*, including lipopolysaccharide (LPS), hemagglutinins, gingipains, and fimbriae, are important in the induction of immune responses, including the production of inflammatory cytokines, such as IL-1, and the activation of inflammation-related signaling pathways [36]. IL-1 also mediates periodontal tissue destruction by stimulating alveolar bone resorption.

Inflammatory and immune mechanisms activated by infectious agents are also important in the development of atherosclerosis [37]. Several epidemiological studies have revealed that host immune reactions against persistent *infectious* pathogens, including *P. gingivalis*, may promote the development of atherosclerosis [38,39]. In this respect, the Nod-like receptor family, pyrin domain containing 3 (NLRP3) inflammasome may play an important role in IL-1β production in response to several bacterial ligands such as LPS, peptidoglycan, and bacterial RNA [40,41,42,43]. Peripheral blood mononuclear cell-derived macrophages reportedly express NLRP3 mRNA [44] to release proinflammatory IL-1β, which is highly induced by bacterial LPS [45]. In contrast, NLRP3-deficient macrophages do not produce active IL-1β in response to bacterial infection [40,41].

NLRP3 proteins were also upregulated in gingival tissues from periodontitis patients [46] and in the aorta of coronary atherosclerosis patients [47]. The oral challenge of ApoE^shl^ mice with *P. gingivalis* showed significantly increased gingival and aortic NLRP3; pro-IL-1β, pro-IL-18, and pro-caspase-1 mRNA levels; and IL-1β, IL-18, and TNF-α peritoneal protein levels compared with gingipain- or fimbriae-deficient mutants, suggesting that gingipains and fimbriae are important virulence factors in NLRP3-inflammasome activation followed by inflammatory cytokine production [48]. Indeed, major fimbriae from *P. gingivalis* induced inflammatory cytokine production by murine peritoneal macrophages and monocytes [49]. Furthermore, gingipains from *P. gingivalis* induced proinflammatory cytokine production in human macrophages [50]. On the other hand, a previous report indicated that NLRP3-related proteins were not secreted into the culture medium due to degradation of the proteins by *P. gingivalis* protease, even if their expression was elevated [51]. However, sufficient levels of IL-1β and TNF-α were produced in cultured murine macrophages by repeated infection with *P. gingivalis* *in vivo* [48]. Because the *in vitro* culture of macrophages is performed using a closed system and an extended culture period, the direct effect of proteases may not reflect *in vivo* competence. In fact, elevated levels of IL-1β can be found in diseased periodontal tissue and gingival crevicular fluid as well as in saliva [46,52,53].

## 4. Activation of the Inflammasome by Oxidative Stress and oxLDL

Host cells produce ROS as a mechanism to combat intracellular microorganisms and as a signal mediator in the cells for various cellular functions [54,55,56]. However, persistent production of ROS causes induction of oxidative stress and may trigger cell death, inflammatory responses, and perturbation of tissue homeostasis in sequence [57]. Recent studies on chronic infections revealed that cytosolic ROS and oxidative-stress pathways play a role in the host metabolic pathway by modulating immune response and persistent infections in specialized immune cells [58,59,60,61].

Signaling by ROS also drives inflammasome activation. ROS activates the inflammasome through mitogen-activated protein kinases and extracellular signal-regulated protein kinases 1 and 2. Dysregulation of the inflammasome plays a significant role in various pathological processes. Recent animal studies show that ROS act as second messengers whose signaling drives NLRP3 inflammasome activation [62,63]. Furthermore, ox-LDL also induces secretion of IL-1b by human macrophages via ROS-dependent NLRP3 inflammasome activation [64]. Therefore, it is possible that the NLRP3 inflammasome is activated through ROS and ox-LDL produced due to infection, as well as through direct inflammasome activation due to infection by periodontal bacteria.

## 5. Persistence of Inflammation by Inflammasome and T helper 17 (Th17) Cell Activation

Activation of the inflammasome is critical for IL-1-driven inflammation and plays a central role in the pathology of autoinflammatory diseases. Recent studies have also shown that IL-1 or IL-18 can promote IL-17-prduction from Th17 cells and γδT cells. In general, IL-1-driven IL-17 production plays a critical role in host protective immunity to infection by fungi, bacteria, and certain viruses. However, in some settings, the sustained production of IL-17 from Th17 cells and γδT cells activated by inflammasome-derived IL-1 or IL-18 have major pathogenic roles in many autoimmune and chronic inflammatory diseases [65].

The presence of IL-17 has been demonstrated in human and experimental atherosclerotic tissues and plasma, and IL-17 might play a role in the pathogenesis of atherosclerosis [66,67]. For example, several studies have reported that IL-17 inhibition via the neutralization of antibodies or target-gene knockdown reduced atherosclerotic plaques in mice [66,68]. When *P. gingivalis*-challenged mice showed notable accumulation of atherosclerotic plaques by Oil Red O staining, the intracellular cytokine staining of spleen cells revealed significantly elevated CD4^+^ IL-17A^+^ T cells and slightly increased CD4^+^ Foxp3^+^ T cells, but the ratio of CD4^+^ interferon gamma (IFN-γ)^+^ T cells hardly changed [69]. IL-17 and IL-1β production and retinoic acid related orphan receptor γt (RORγt) expression were also significantly increased in the splenic cells of *P. gingivalis*-challenged mice. Furthermore, the expression of Th17-related genes, such as IL-6, TGF-β, RORγt, signal transducer and activator of transcription 3 (STAT3), and IL-23, were elevated in the splenic cells and heart tissue of *P. gingivalis*-challenged mice. These results suggest the possibility that ROS or ox-LDL formed due to infection by *P. gingivalis* activates Th17 cells through the NLRP3 inflammasome. Indeed, Lim *et al*. [70] reported that ox-LDL contributes to the development of Th17 in murine arteriosclerosis.

Recent studies have also demonstrated that *P. gingivalis* promotes Th17-inducing pathways in chronic periodontitis [71,72]. Persistence of the Th17 population has been shown to support chronic inflammation and to directly mediate tissue destruction through the activation of resident matrix cells such as fibroblasts and osteoclasts *in vitro* [73,74]. Therefore, uncovering the mechanisms through which *P. gingivalis* may contribute to the generation of chronic inflammatory tissue-destructive mechanisms may be relevant not only for periodontitis but also for several systemic inflammatory diseases such as atherosclerosis.

Serotype b *Aa* challenge also increased the murine splenic IL-17^+^CD4^+^ T cell population, Th-17-related serum cytokine levels (IL-17, IL-6, TGF-β, and IL-1β), Th17-related gene expression (IL-1β, IL-6, IL-17RA, STAT3, IL-21, and TGF-β), and atherosclerotic plaque formation in the aorta [75]. Furthermore, the *Aa* challenge increased the expression of Th17-related microRNA (miR-155 and miR146b) and the inflammasome-related molecules absent in melanoma 2 (AIM-2), Mincle, and NLRP3 in the aorta. These results suggest the possibility that the increase in Th17 cells observed in *Aa*-challenged mice is related to exacerbation of atherosclerosis. Serotype b *Aa* strains have been shown to have a greater capacity to trigger Th17-type cytokine production *in* *vitro* [76]. Serotype b strains are more frequently detected in patients with chronic and aggressive periodontitis than in healthy subjects [77,78]. Therefore, the association between the presence of serotype b *Aa* strains and periodontitis suggests the increased inflammatory potential of this species. 

Because *Aa* challenge significantly increased AIM-2, Mincle, and NLRP3 mRNA expression in ApoE^shl^ mice, recognition of innate and inflammatory signals by dendritic cells via pattern-recognition receptors may activate the intracellular pathways involved in CD4^+^ T cell differentiation into Th17 cells.

It was recently shown that miR-155 was expressed in mouse and human atherosclerosis lesion [79]. Further, miR-155 deficiency in Apolipoprotein E knockout (ApoE-KO) mice attenuated atherosclerosis by reducing macrophage inflammation and Th17 cell numbers. Indeed, miR-155 promotes the development of inflammatory Th17/Th1 cell subsets [80]. MicroRNA (MiR)-146b was also shown to be involved in the pathogenesis of murine viral myocarditis and *Clostridium difficile*-related colitis by regulating Th17 differentiation [81,82]. As *Aa* challenge significantly increased miR-155 and miR-146b microRNA expression in ApoE^shl^ mice, *Aa* may regulate Th17 differentiation by enhancing miR-155 and miR-146b expression [75].

## 6. Prevention of Periodontal Disease and Atherosclerosis Using Antioxidants

A positive correlation between green tea consumption and cardiovascular health has been established in cohort or cross-sectional study [83]. Catechins are the main polyphenolic compounds in green tea. They exert vascular protective effects through multiple mechanisms, including anti-inflammatory, antioxidant, antihypertensive, anti-thrombogenic, and lipid lowering effects, that are effective in vascular protection [84]. Catechins perform antioxidant activity by scavenging free radicals, inhibiting redox active transcription factors, inhibiting pro-oxidant enzymes, and inducing antioxidant enzymes. They are effective in attenuating lipid biosynthesis via modulation of relevant enzymes and regulation of intestinal lipid absorption, thereby improving serum lipid profiles. These effects are critical in influencing the progression of atherosclerotic lesions and amelioration of cardiovascular diseases with improved endothelial function [85]. Atherosclerotic lesion areas of the aortic sinus caused by *P. gingivalis* infection decreased in epigallocatechin-3-gallate (EGCG)-treated ApoE-KO mice, wherein EGCG reduced the production of C-reactive protein, MCP-1, and oxidized LDL and slightly lowered LDL/very low-density lipoprotein (VLDL) cholesterol in *P. gingivalis*-challenged mice serum [86]. Furthermore, the increases in inflammatory- and oxidative stress-related mediators, such as chemokine (C-C motif) ligand 2 (CCL2), MMP-9, intercellular adhesion molecule-1(ICAM-1), heat shock protein 60 (HSP60), CD44, lectin-like receptor-1 for ox-LDL (LOX-1), NADPH oxidase 4 (NOX-4), p22phox, and inducible nitric oxide synthase (iNOS) gene expression levels in the aorta of *P. gingivalis*-challenged mice were reduced by EGCG-treatment. However, HO-1 mRNA levels were elevated by EGCG treatment, suggesting that EGCG inhibits *P. gingivalis*-induced atherosclerosis through anti-inflammatory and anti-oxidative effects [86]. Therefore, previous catechin consumption may be useful for the prevention of pathogen-accelerated atherosclerosis.

Positive correlation is also obtained application of antioxidant to periodontitis. Topical application of a dentifrice containing green tea catechin in an induced periodontitis rat model also reduced inflammatory cell infiltration in the periodontal lesions to a greater extent than did the control dentifrice [87]. Gingival expression of a lipid peroxidation marker hexanoyl-lysine, nitrotyrosine, a marker of oxidative protein damage, and TNF-α was reduced in response to the dentifrice with green tea catechin compared with the control dentifrice [87]. Therefore, addition of green tea catechin to a dentifrice may prove useful for the improvement of periodontal inflammation by reducing oxidative stress and the expression of pro-inflammatory cytokines. Recently, it was shown that catechin suppressed LPS-induced inflammatory bone resorption, and protected against alveolar bone loss in mice [88]. Catechin may be effective in preventing gingival and periodontal inflammation and bone loss. Several proteolytic enzymes, including gingipains, collagenases, and dipeptidyl aminopeptidase, produced by *P. gingivalis* also play important roles in the pathogenesis of periodontitis by hydrolyzing serum and tissue proteins, leading to tissue destruction [89]. Therefore, inhibitors of these enzymes may play important roles as valuable therapeutic agents. Polyphenols isolated from cranberry and green tea, which are natural plant-derived inhibitors of *P. gingivalis* proteases, are also effective in the inhibition of several proteases produced by *P. gingivalis* [89]. Furthermore, the epidemiological relationship between the intake of green tea and periodontal disease also demonstrated a significant reduction in periodontal pocket probing depths [90] and tooth loss [91]. These results indicate an association of catechin consumption with reduced odds for probing depths and tooth loss. A recent clinical trial also indicated that local application of green tea catechin gel in chronic periodontitis reduced periodontal pockets and inflammation [92]. Furthermore, locally delivered lycopene gel as an antioxidant in the treatment of chronic periodontitis was effective in increasing clinical attachment and reducing gingival inflammation, probing depth, and oxidative injury [93].

## 7. Conclusions

That oxidative stress and ox-LDL levels were increased in the blood and local sites of periodontal or atherosclerotic patients and in the mouse model suggests that lipid peroxidation activates the NLRP3 inflammasome and contributes to the activation of IL-17-producing Th17. Furthermore, it suggests the possibility that infection and sustained production of inflammatory cytokines, including IL-1β, IL-18, and IL-17, through lipid peroxidation results in chronic inflammation. On the other hand, treatment of catechin, a polyphenol with antioxidant, anti-inflammatory, and antithrombotic activities, protected mice from infection caused by periodontopathic bacteria and suppressed gingival inflammation, bone resorption, and arteriosclerotic progress. These results confirm the role of lipid peroxidation, as a correlation between periodontal disease and atherosclerosis, and can be a starting point for further research on the efficiency of different antioxidant agents in the prevention and treatment of periodontal disease and atherosclerosis. However, there are several limitations to the present study. The level of oxidative stress is a result of the balance between ROS production and anti-oxidant defense [94]. Although ascorbic acid and α-tocopherol are low-molecular-mass anti-oxidants that protect the cells from ROS and are a part of anti-oxidant defense system [95], their low concentration is associated with a higher risk of several chronic inflammatory diseases that are related to periodontal disease [96], diabetes [97], and coronary heart disease [98,99]. Increased plasma glutathione peroxidase concentration in GCF, which is an antioxidant in the human defense against oxidative stress, can also be considered as an indicator of a local increase in oxidative stress [100]. Therefore, the increase in ROS generation may have led to the occurrence of oxidative stress, which in turn caused an increased need for antioxidant production to establish the ROS-anti-oxidants balance to protect the tissues. Further study with larger samples and more complete periodontal health and disease information would be necessary to substantiate these findings.
